# A One Health Perspective on Multidrug Resistance Amongst Iberian Exotic Pet Owners

**DOI:** 10.3390/vetsci12010064

**Published:** 2025-01-16

**Authors:** Fábio Cardoso-Freitas, Stéphanie M. Mota, Vanessa Silva, Albert Martinez-Silvestre, Ângela Martins, Patrícia Poeta

**Affiliations:** 1 Veterinary and Animal Research Centre (CECAV), University of Trás-Os-Montes and Alto Douro (UTAD), 5000-801 Vila Real, Portugal; al53178@alunos.utad.pt (F.C.-F.); angela@utad.pt (Â.M.); 2 Veterinary Department, Royal Zoological Society of Scotland, Edinburgh Zoo, Edinburgh EH12 6TS, UK; 3 MicroART—Microbiology and Antibiotic Resistance Team, Department of Veterinary Sciences, University of Trás-Os-Montes and Alto Douro (UTAD), 5000-801 Vila Real, Portugal; 4 Associate Laboratory for Green Chemistry (LAQV-REQUIMTE), University NOVA of Lisboa, 1099-085 Lisboa, Portugal; 5 Department of Genetics and Biotechnology, University of Trás-Os-Montes and Alto Douro (UTAD), 5000-801 Vila Real, Portugal; 6 Functional Genomics and Proteomics Unit, University of Trás-Os-Montes and Alto Douro (UTAD), 5000-801 Vila Real, Portugal; 7 Catalonian Reptiles and Amphibians Rescue Center (CRARC), 08783 Masquefa, Barcelona, Spain; crarc-masquefa@outlook.com; 8 Associate Laboratory for Animal and Veterinary Sciences (AL4AnimalS), 5000-801 Vila Real, Portugal

**Keywords:** non-traditional companion animals, antibiotic, antimicrobial, parasiticide, deworming, zoonoses, Portugal, Spain

## Abstract

Understanding owners’ realities and perceptions may be crucial to helping veterinarians be more effective in their daily work. Noticing the lack of publications in this area, specifically on exotic pets, prompted us to gather insights into non-traditional companion animal owners. A survey was conducted, resulting in 541 respondents from Portugal and Spain. The results were characterised based on demographics, data on trade and antibiotic and parasiticide use, and the relations between these variables. We found that there is still a considerable part of unregistered animals and that, overall, owners acknowledged antimicrobial resistance and inadequate use of parasiticides. Hopefully, these data will help veterinarians in tackling these problems and enhance owners’ education and compliance.

## Simple Summary

Understanding owners’ realities and perceptions may be crucial to helping veterinarians be more effective in their daily work. So, we aimed to gather the insights of non-traditional companion animal owners in Portugal and Spain on trade, zoonosis, and antibiotic and parasiticide use. We found that there is still a considerable part of unregistered animals and that, overall, owners acknowledged antimicrobial resistance and inadequate use of parasiticides. Hopefully, these data will help veterinarians in tackling these problems and enhance owners’ education

## 1. Introduction

### 1.1. Non-Traditional Companion Animals

The wide range of species kept as pets, excluding domestic animals (such as dogs, cats, cows, horses, pigs, or sheep) that may be native or non-native, can be defined as non-traditional companion animals (NTCAs), or more colloquially, exotic pets [1,2]. The number of exotic pets has been increasing [2,3,4,5,6], and younger generations are more likely to be interested in keeping these pets [7].

According to the latest companion animal statistics, the combined population in Portugal (PT) and Spain (SP) was 9,753,000 NTCAs, including 5,701,000 birds, 782,000 aquaria, 1,762,000 small mammals, and 1,508,000 reptiles, making up 32.8% of the combined total pet population [8]. There are no data regarding the prevalence of owning these animals. Also, no official data were given from the governments of both countries.

### 1.2. Animal Trade

In the absence of clear guidelines for animal trade, the International Union for the Conservation of Nature’s (IUCN) Red List of Threatened Species and the Convention on International Trade in Endangered Species of Wild Fauna and Flora (CITES) Appendices have been adopted as the basis for negative list trade restrictions [9]. However, these lists do not cover all species that are vulnerable since their exploitation varies with market trends [9,10]. Therefore, the trading and keeping of NTCAs is a subject of current discussion; for example, SP is working on a positive listing policy while PT maintains a negative listing [11,12].

The demand for NTCAs is a crucial driver for global trade [6]. Initially, most organisations aimed to conserve native biodiversity at the origin of the trade. However, it is now equally important to fight the emergence of invasive species at the end of the trade, which have been endangering local species and habitats for years [13,14,15,16]. This further complicates the current regulations, as the exotic pet trade can shift from legal to illegal, or both, as borders are crossed between nations and policies [13,14].

With Europe being one of the most important markets for the illegal wildlife trade and trafficking, it is reasonable to assume that efforts should be made to simplify regulations, namely a standardised definition for domestication [13,17,18].

### 1.3. Veterinary Care, Communication, and Medical Information

Pasteur et al. defined access to veterinary care as “the economic, physical, social, mental, and emotional resources necessary for people to secure, communicate with, and benefit from the services of a trusted veterinary service provider are available as needed to optimise the health and welfare of animals in their care” [19]. For example, if an animal is maintained illicitly, his caretaker is less likely to reach out to a veterinarian, negatively impacting the animal’s welfare [13].

Since veterinarians are considered reliable sources of information by owners [19,20,21], training these professionals in communication has become of major importance so that they can fulfil the expectations of owners and use the current means of communication to reach the public [22,23]. This training can be crucial in helping to deconstruct general misconceptions about pet husbandry, behaviour, and welfare [24].

### 1.4. Zoonotic Risks

Before the COVID-19 pandemic, the demand for them [6]. That same study proposed using this information as a foundation to educate consumers to do their research before purchasing animals and to do so in the proper places [6]; this would promote animal welfare and reduce zoonotic risks [24,25]. But even after COVID-19, it is necessary to continue educating the general public about zoonoses because most owners were not at all concerned about the risk of zoonotic transmission with their pets [21]. Notably, studies conducted in PT found that between 56.5% and 85% of respondents had heard about “zoonosis” [26,27], and another study specified that 67.8% of adolescents were aware of what zoonosis means. In comparison, only 35.2% of adults know the same meaning, showing a significant lack of knowledge of biological risks and zoonoses [20,26,27]. The pros and cons of the human–companion animal relationship have already been described, and keeping NTCAs is associated with a higher zoonotic risk [3]. Detailed information on zoonotic risks, specific to each taxon, already exists [28,29,30,31,32,33,34,35]. Thus “veterinarians must not only treat animals with zoonosis but also play a role in the prevention of zoonosis in humans”, by providing education and preventive measures to reduce the probability of zoonosis transmission [36].

### 1.5. Antibiotics

The transmission of bacteria and antimicrobial resistance (AMR) between humans and pets is bidirectional [37]. Although additional studies are still needed on the NTCA AMR specifically, there are already Iberian studies [38,39,40], and the presence of AMR in NTCA feed has also been described [41]. Pets can act as reservoirs of bacteria, so education is a key element for reducing the associated zoonotic risk [42,43].

Owners of sick pets have an important appeal to the use of antibiotics (ABs), and understanding the risks of the broader use of ABs depends on the owner’s health literacy and consultation time [44]. A more straightforward and preventive approach would be to improve animal welfare, which could consequently reduce the usage of ABs [45]. Besides prudent AB use, the owner’s compliance with the therapy is critical. The consequences of using ABs without a veterinary prescription and the early discontinuation of the therapy are well known and of concern [46,47]. But no studies on Iberian NTCA owners have been published. Reducing the frequency and using sustained-release medications may help in cases where there are no practical therapeutic options for NTCAs [23].

### 1.6. Parasiticides

NTCAs comprise several species, so it is to be expected that they also harbour numerous parasites, which pose a zoonotic concern [48,49,50,51].

There is a demand for responsible use of parasiticides or anti-parasitic drugs (APs) in companion animals [52]. However, concerns about resistance due to misuse/overuse are already known in other veterinary sectors [52,53]. Besides the risk of resistance, APs also have an impact on the environment, displaying serious evidence of ecotoxicity [54,55,56]. The indications are to avoid blanket treatments, to tailor the treatment to each individual and the risks they are exposed to, and to carry out routine diagnostics instead [48,52,57].

A European study on deworming practices in dogs and cats showed that treatment compliance needed to be improved, and similar results were observed in studies made in PT and SP [26,58,59]. In the Iberian studies, veterinarians were encouraged to engage caretakers more frequently in prevention instructions, thus increasing public awareness [26,59]. But no studies have been published on NTCAs.

Looking through the owner’s perspective could help veterinarians provide more targeted education. In a study of dog owners, the veterinarian’s recommendation was one of the main factors in choosing APs, along with a broad spectrum of action and the price [60].

### 1.7. Aims of This Study

The common ground in the previously discussed topics was that no studies have been published regarding NTCA owners. Hence, the primary aim of this survey was to investigate the opinions, preferences, and knowledge of NTCA owners with respect to aspects related to veterinary consultation such as zoonosis, antibiotic and parasiticide usage, and the associated risks. The secondary aim was to determine whether there were differences between demographic groups. This would increase the effectiveness of the communication between veterinarians and NTCA owners, thus providing better care for NTCAs.

## 2. Material and Methods

After initial investigations on similar subjects, we developed a questionnaire with 64 questions written in Portuguese and Spanish, using Google Forms to collect NTCA owners’ opinions. We used an image with a QR code, and a brief description (including a short link to the survey) was shared through email (to Zoological Medicine veterinarians and exotic animal associations or retailers) and social media (Facebook groups and exotic animal Instagram pages). Participation rights were assured, namely voluntary participation, through anonymity, informed consent, and data confidentiality, following the applicable regulations (Declaration of Helsinki, Oviedo Convention, General Data Regulation, and the European Code of Conduct for Research Integrity ESF/ALLEA).

To be eligible, participants had to meet the following criteria: groups of people aged 18 years and over, NTCA owners, living in Portugal or Spain, and completed the online survey from 29 January to 24 April 2024. All statistical analyses and descriptions were performed using Excel 16.86 and JMP^®^ version 17.2.0. The Chi-square (X^2^) or Fisher’s exact tests were used where appropriate, and statistical significance was considered when *p* < 0.05.

We established a population of 49,841,263 possible respondents based on the data of major age groups from the two countries combined [3,4], as there are no numbers regarding NTCA owner prevalence for these countries. A confidence interval of 95% and an estimated true proportion of 0.1 were based on a study carried out in Ireland [2]. We used the EPITOOLS epidemiological calculator and estimated a minimum sample of 139 answers.

## 3. Results

### 3.1. Demographics

**Figure 1:**
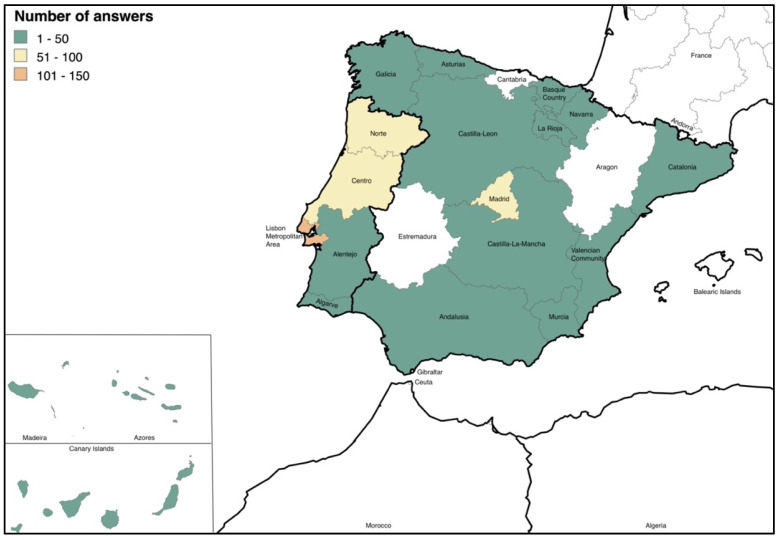
Distribution by number of answers from owners according to NUTSII (created with mapchart.net).

The number of respondents was 548; however, 7 respondents were excluded: 5 for being from other countries (Brazil, England, France, United Kingdom, and Switzerland) and 2 for being underaged. Therefore, the responses of 541 respondents were included in this study (PT = 367; SP = 174). To be clearer, we also refer to respondents as NTCA owners, caretakers, or keepers. Figure 1 details the respondents’ location distributions. Additionally, 22.2% of the respondents were from rural areas, while 77.8% were from urban areas, with no significant difference between PT and SP.

**Figure 2:**
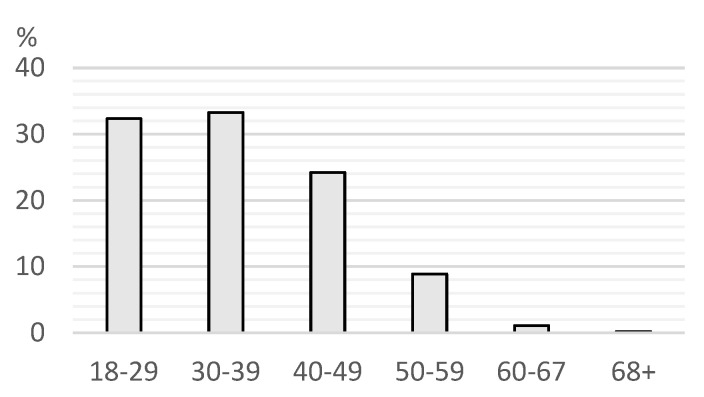
Percentage of respondents according to intervals of ages.

Regarding gender, 75.6% were female, 24% were male, and 0.4% were nonbinary. The distribution of ages is detailed in Figure 2.

**Figure 3:**
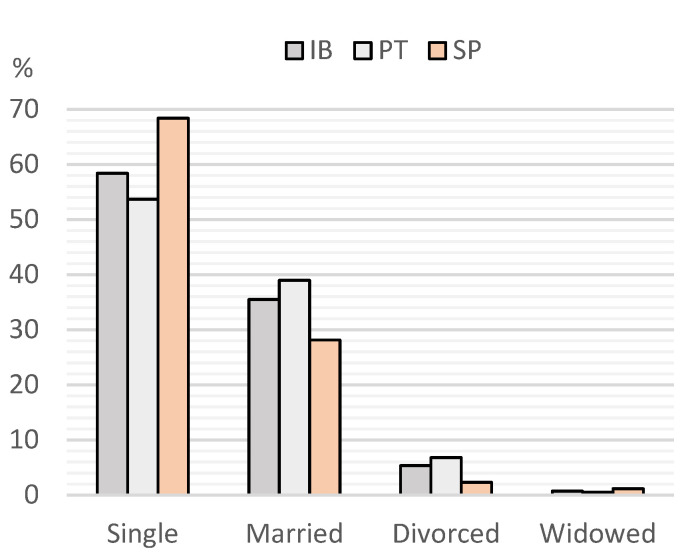
Percentage of respondents according to their marital status. IB—Iberia; PT—Portugal; SP—Spain.

Additionally, 1.3% identified themselves as low social class, 15.2% as middle class, and 83.5% as upper class; 80% were employed, but there was a significant difference between countries where Portuguese respondents were 1.7 times more likely to be employed (PT 83%, SP 74%, *p* = 0.02). The marital status detailed in Figure 3 showed a significant difference between PT and SP (*p* = 0.004), and 34% reported having children.

**Figure 4:**
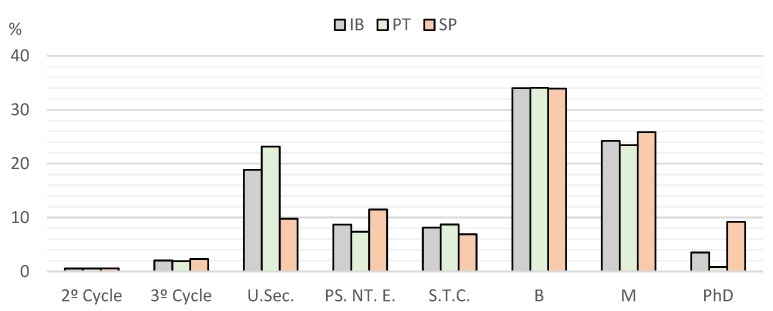
Distribution of respondents according to their level of education. IB—Iberia; PT—Portugal; SP—Spain; U.Sec.—upper secondary; PS. NT. E.—post-secondary non-tertiary education; S.T.C.—specialised technological course; B—bachelor’s; M—masters; PhD—doctorate.

When asked about their education level, summarised in Figure 4, data showed a highly significant difference between the countries (*p* < 0.0001).

### 3.2. NTCA Numbers and Trade

**Figure 5:**
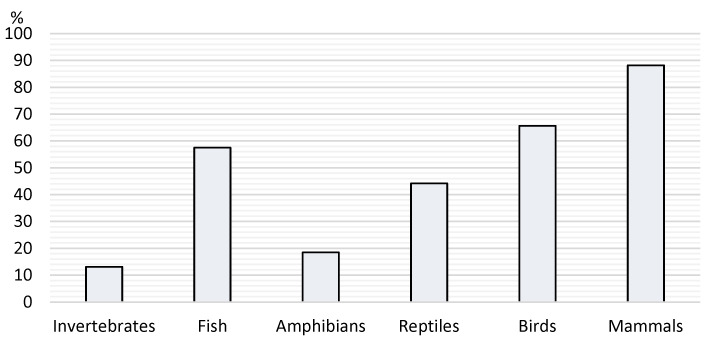
Prevalence of animals kept by the respondents.

Of all the respondents, 12.8% were breeders (65.2% were registered breeders), and 6.3% were sellers of NTCAs (47.8% were also breeders). By asking what animals the respondents kept, we determined an idea of their prevalence, detailed below in Figure 5.

There was a difference when comparing breeders and sellers with the remaining respondents. Breeders were 3.5 times more likely to keep amphibians and birds (2.0|6.1 *p* < 0.0001; 1.7|7.0 *p* = 0.0001), 4.4 times more likely to keep invertebrates (2.5|7.9 *p* < 0.0001), and 2.7 times more likely to keep reptiles (1.6|4.6 *p* = 0.0002). Sellers had 4.5-fold greater odds of keeping amphibians (2.2|9.1 *p* < 0.0001), 3.2-fold greater odds of keeping birds (1.2|8.5 *p* = 0.0142), 6.4-fold greater odds of keeping invertebrates (3.1|13.2 *p* < 0.0001), 2.5-fold greater odds of keeping fish (1.1|5.7 *p* = 0.0205), and 2.8-fold greater odds of keeping reptiles (1.3|5.9 *p* = 0.0067). A total of 81% of the NTCA keepers investigated the species’ needs before acquiring them, with no significant difference between countries.

Concerning the CITES appendixes, 59% of respondents were aware of it, and 43% reported they had the appropriate documentation when acquiring the animals. Additionally, 41% of respondents knew what positive lists were. There was no significant difference between PT and SP, but there was a difference when comparing breeders and sellers with the remaining respondents. Breeders were 5.9 times more likely to investigate before acquiring the animals (1.8|19.2 *p* = 0.0004), 7.4 times more likely to know what CITES was (3.3|16.5 *p* < 0.0001), 7.2 times more likely to have the proper documentation of acquisition (3.9|13.6 *p* < 0.0001), and 4.5 times more likely of know what positive listing was (2.6|7.9 *p* < 0.0001). Sellers had 8.3-fold greater odds of investigating prior to the acquisition of the animals, 25.5-fold greater odds of knowing what CITES was (3.5|187.9 *p* < 0.0001), 6.9-fold greater odds of having the proper transaction papers (3.8|17.0 *p* < 0.0001), and 9.3-fold greater odds of knowing what positive listing was (3.5|24.4 *p* < 0.0001).

### 3.3. Veterinary Care

Regarding the questions about the knowledge of Zoological Medicine (ZM) veterinarians, 96.7% of the respondents were aware of the existence of these professionals. While 98.5% knew of their existence in the country, 81.3% knew of their existence in their area, and 39.6% had consulted one. Keepers of mammals were 13.4 times more likely to be aware of ZM veterinarians in the country (3.1|57.5 *p* = 0.0008) and invertebrate keepers were 1.8 times more likely to have attended a ZM appointment (1.1|3.0 *p* = 0.0264).

### 3.4. Zoonotic Risks

Of all the respondents, 31% were human/animal health professionals, and 60% recognised zoonosis. More details on the influence of various variables on the identification of the term zoonosis can be found in Table 1.

Health professionals had 13 times greater odds of identifying the term, with 92% saying they knew what zoonosis was versus 46% of respondents who were not health professionals and had the same knowledge.

### 3.5. Antibiotics

**Figure 6:**
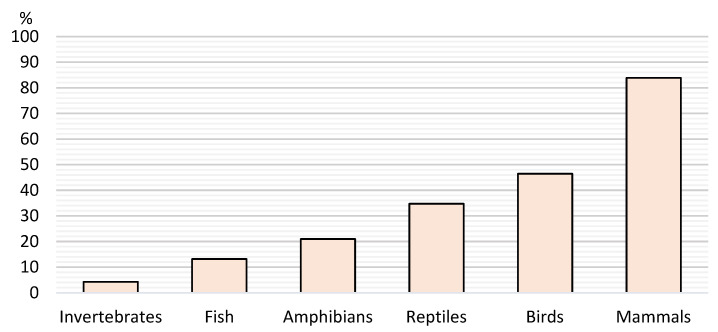
Prevalence of antibiotic usage according to determinate groups of animals.

When it comes to antibiotics, 97% of the respondents said that these drugs were used against bacteria, with the people who know what zoonosis is being 5 times more likely to say this (1.7|16.0 *p* = 0.004). Additionally, 24% of the respondents said that ABs are used against fungus, with people from lower social classes and aged between 18 and 29 years old being twice as likely to say so (1.3|3.5 *p* = 0.0045 and 1.3|2.9 *p* = 0.0024, respectively). Also, 23% said that ABs are used against parasites, with respondents aged between 18 and 29 years old having almost twice the odds of affirming it (1.1|2.6 *p* = 0.0118). Finally, 24% of the respondents said that ABs are used against viruses with those with a specialised technological course having two-fold greater odds of saying so (1.4|3.6 *p* = 0.0017). By asking the participants which animals they used ABs for, we obtained the data compiled in Figure 6.

Of all the participants of the study, 97% were aware of AMR, with respondents who know what zoonosis is having 8 times greater odds (2.3|28.0 *p* = 0.0003) and mammals’ caretakers having 5 times greater odds of being aware of said resistances (1.9|14.0 *p* = 0.0026). Eighty-five percent knew that AMR is linked to a share of human deaths, with respondents who recognise the term zoonosis being up to 2.6 times more likely to acknowledge that (1.6|4.2 *p* < 0.0001), health professionals being 3.7 times more likely to recognise it (1.8|7.4 *p* < 0.0001), and respondents who were aware of AMR being 10.6 times more likely to acknowledge it (3.7|30.1 *p* < 0.0001). About 60% of participants thought that the ABs could harm their animals, and health professionals and respondents familiar with the term zoonosis were almost twice as likely to think so (1.2|2.7 *p* = 0.0024 and 1.2|2.4 *p* = 0.0041). The influence of recognising AMR and other selected variables on said use is detailed in Table 2.

Respondents were questioned whether their veterinarian had ever explained to them what an antibiotic sensitivity test (AST) was, and 46% of the respondents replied positively. Also, 65% said that they accepted this test when proposed by their veterinarian. The influence of the chosen variables is detailed in Table 3.

When asked whether veterinarians should be the ones to decide when animals need ABs, 99% answered positively. Moreover, 97% of owners considered that the frequent use of ABs tends to decrease their efficacy in the future, with owners who were aware of AB resistance being 7 times more likely to feel this way (1.9|28.0 *p* = 0.0157). A total of 2% of respondents believe that their animals need ABs when they are sick. When an animal shows clinical signs similar to those that left them to take ABs previously, 5% of respondents think they can give the animals ABs again, with low-social-class respondents being almost 3 times more likely to think that (1.2|6.6 *p* = 0.025) and breeders or sellers being 3.6 times more likely to also believe that (1.5|8.3 *p* = 0.005 and 1.3|10.2 *p* = 0.0249, respectively). A total of 21% of caretakers felt they knew when their animals needed ABs, with the odds of this feeling being 1.7-fold greater for Spanish caretakers (1.1|2.5 *p* = 0.0238), 1.8-fold greater for caretakers with a bachelor’s degree (1.1|2.8 *p* = 0.0126), 3.8-fold greater for breeders (2.2|6.5 *p* < 0.0001), 4.8-fold greater for sellers (2.4|9.8 *p* < 0.0001), 3.6-fold greater for health professionals (2.3|5.5 *p* < 0.0001), 2.0-fold greater in caretakers who know what zoonosis is (1.3|3.2 *p* = 0.0018), 6.8-fold greater for owners who used ABs in amphibians (3.0|15.5 *p* < 0.0001), 1.9-fold greater for owners who used ABs in birds (1.2|2.9 *p* = 0.0047), 2.2-fold greater for owners who used ABs in fish (1.1|4.2 *p* = 0.0304), 2.8-fold greater for owners who used ABs in mammals (1.5|5.2 *p* = 0.0005), and 2.6-fold greater for owners who used ABs in reptiles (1.6|4.3 *p* = 0.0004).

When analysing the use of ABs, the results show that 6% of caretakers used ABs without consulting a veterinarian, with breeders and sellers being 10 times more likely to do so (5.2|22.4 *p* < 0.0001 and 5.0|26.0 *p* < 0.0001, respectively), caretakers who used ABs in fish being 3 times more likely to do so (1.3|8.0 *p* = 0.0151), and caretakers who used ABs in birds being 6 times more likely to do so (2.9|13.0 *p* < 0.0001). The source of ABs used without a prescription was 36% from leftovers, 19% online without a prescription, 19% from family or friends, 17% from a pharmacy without a prescription, and 6% from a pet shop without a prescription. Also, 97% of caretakers completed the course of ABs prescribed, with those who acknowledge AB resistance having an 8 times greater chance of doing so (2.0|30.4 *p* = 0.0132), as well as mammals’ caretakers who were also 3.5 times more likely to do so (1.2|10.7 *p* = 0.0308). The respondents who stated they had not completed a course of ABs explained their reasons; namely, 36% thought animals did not need the entire course and 18% of respondents stated it was because the animal improved quickly, 18% because they ran out of medication, 18% because of an adverse reaction, and 9% because the animal rejected the treatment.

A small percentage of respondents revealed they had stopped seeing a veterinarian because they did not prescribe an antibiotic (1%). On the other hand, 8% think that the prescription of antibiotics is a way for veterinarians to make more money. Breeders are twice as likely to think that a phone call is enough for veterinarians to know if an animal needs ABs (1.1|4.6 *p* = 0.0388), but overall, 9% of all respondents think this. Furthermore, 27% of participants feel that ABs should be prescribed at the beginning of the illness and veterinarians should not wait for developments. Still, 24% of all caretakers feel safer if an AB is prescribed when their animal is sick, and 35% feel safer if a broad-spectrum antibiotic is prescribed, with caretakers with a master’s degree being almost two times more likely to feel so (1.2|2.6 *p* = 0.0048).

### 3.6. Parasiticides

**Figure 7:**
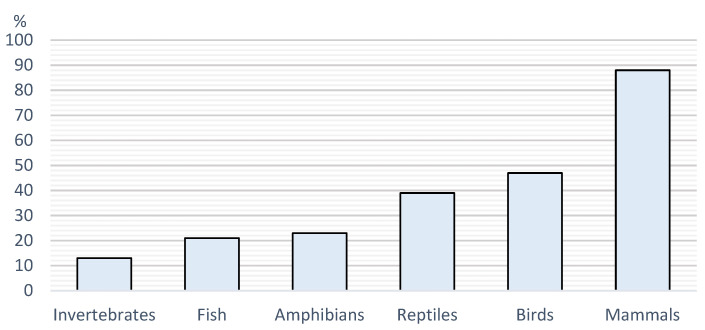
Prevalence of parasiticide usage according to determinate groups of animals.

Regarding APs, 98% of respondents stated that they were used against parasites, 7% stated that they were used against bacteria, 14% stated that they were used against fungus, and 3% stated that they were used against viruses. Caretakers from rural areas were twice as likely to state that APs were used against fungus (1.2|3.6 *p* = 0.007) and three times more likely to state that APs were used against viruses (1.1|7.6 *p* = 0.0374). The answer to the question of in which animals respondents used APs is detailed in Figure 7.

Almost 79% of the respondents recognised the inappropriate use of parasiticides, and 48% admitted that it occurs in their country, with no significant difference between countries. Moreover, 39% thought that the use of APs may hurt their animal, and 55% thought that the frequent use of APs may lead to decreased efficacy in the future. The influence of recognising the inappropriate use of APs and other selected variables on this use is detailed in Table 4.

Respondents were also questioned whether their veterinarian had ever explained to them what a coprological analysis was, with 77% saying yes. Also, 80% of respondents stated that they accepted the coprological analysis when proposed by their veterinarian. The influence of chosen variables is detailed in Table 5.

On the other hand, 67% of owners said their veterinarian had explained to them the risks and benefits of using APs according to the animal’s lifestyle. The likelihood of owners and veterinarians having this conversation was as follows: 1.6 times higher for owners in their 40s (1.0|2.5 *p* = 0.0431), 2.3 times higher for health professionals (1.5|3.4 *p* < 0.0001), 2.5 times higher for owners that acknowledge zoonosis (1.7|3.6 *p* < 0.0001), 3.9 times higher for owners who recognised inappropriate use of APs (2.5|6.0, *p* < 0.0001), 4.0 times higher for owners who knew what coprological analysis was (2.6|6.1, *p* < 0.0001), 2 times higher for owners who gave APs to birds, fish, and reptiles (1.1|2.4 *p* = 0.0245, 1.1|3.7 *p* = 0.0258 and 1.1|3.2 *p* = 0.0127, respectively), and 4.7 times higher for owners who gave APs to mammals (2.9|7.4 *p* < 0.0001).

Regarding whom should decide on when pets need APs, 82% of respondents said that it should be the veterinarian, with respondents from the middle class and respondents who were aware of the existence of ZM veterinarians being three times more likely to think so (1.7|4.8 *p* = 0.0001 and 1.2|8.5 *p* = 0.0235, respectively). However, 36% of respondents thought they knew when their animal needs APs, with the odds of thinking that being 2.8 times greater for breeders (1.7|4.7 *p* < 0.0001), 4.1 times greater for sellers (1.9|8.5 *p* = 0.0001), 2.0 times greater for health professionals (1.4|2.9 *p* = 0.0003), 1.7 times greater for respondents that recognised the term zoonosis (1.2|2.4 *p* = 0.0079), 3.8 times greater for respondents that recognised the inappropriate use of APs (2.2|6.5 *p* < 0.0001), 1.8 times greater for respondents who knew what coprological analysis was (1.2|2.9 *p* = 0.0081), 1.5 times greater for respondents who attended a ZM appointment (1.0|2.1 *p* = 0.0351), 3.3 times greater for respondents who used APs in amphibians (1.4|7.7 *p* = 0.0047), 2.0 times greater for respondents who used APs in birds (1.4|2.8 *p* = 0.0005), 2.4 times greater for respondents who used APs in fish (1.4|4.0 *p* = 0.0015), 4.3 times greater for respondents who used APs in invertebrates (1.1|16.7 *p* = 0.0404), 5.2 times greater for respondents who used APs in mammals (2.8|9.8 *p* < 0.0001), and 2.8 times greater for respondents who used APs in reptiles (1.8|4.5 *p* < 0.0001). Of all the respondents, 4% thought their animal needed APs when ill; also, 19% thought that they could give APs when their animals developed the same signs as the last time that an AP was prescribed, with parents having a 1.6-fold greater probability of doing so (1.0|2.5 *p* = 0.0375), the middle class having a 2.2-fold bigger probability (1.3|3.7 *p* = 0.0055), breeders having a 4.6-fold bigger probability (2.7|7.9 *p* < 0.0001), sellers having a 4.4-fold bigger probability (2.1|8.9 *p* < 0.0001), and respondents that used APs in birds, mammals, and reptiles being twice as likely (1.2|3.0 *p* = 0.0046, 1.1|4.1 *p* = 0.0328, and 1.3|3.6 *p* = 0.0039, respectively).

Regarding AP use, 26% of caretakers used them without attending a ZM appointment, with parents being 1.7 times more prone (1.2|2.5 *p* = 0.0133), breeders being 4.3 times more prone (2.6|7.3 *p* < 0.0001), sellers being 3.5 times more prone (1.7|7.0 *p* = 0.0009), caretakers who acknowledged zoonosis being 1.9 times more prone (1.3|2.4 *p* = 0.0019), caretakers who went to a ZM appointment in the past being 1.9 times more prone (1.3|2.7 *p* = 0.0019), caretakers who acknowledged inappropriate use of APs being 1.9 times more prone (1.1|3.2 *p* = 0.0168), caretakers who gave APs to birds being 2.9 times more prone (2.0|4.3 *p* < 0.0001), and caretakers that gave APs to reptiles being 1.8 times more prone (1.1|2.8 *p* = 0.0213). When asked what the sources for the APs were, 52% claimed they obtained it from a pharmacy without a prescription, 22% claimed they obtained it online without a prescription, 14% claimed that they had leftovers from previous use, 7% claimed they obtained it at a pet shop without a prescription, and 3% claimed they obtained it from family or friends.

Concerning AP compliance, 97% of respondents stated they had completed the prescription’s duration, with respondents who gave APs to mammals being 5.4 times more likely to have done so (2.0|14.2 *p* = 0.0012). On the question of whether they felt safer when an AP was prescribed, 23% replied positively. Also, 34% felt safer if a broad-spectrum AP was prescribed. Furthermore, 1% of respondents said they stopped seeing their previous veterinarian because had not been prescribed an AP prescription, with breeders being eleven times more likely to do so (1.8|65.1 *p* = 0.0165) and sellers being twenty-five times more likely to do so (4.0|151.7 *p* = 0.0021). In addition, 39% of respondents stated that an AP should be prescribed at the beginning of the illness and they should not have to wait to see how it develops. Owners that felt that a veterinarian should know when to prescribe an AP from a phone call comprised 18% of all respondents, with parents, breeders, and bird and amphibian caretakers being twice as likely to feel so (1.3|3.1 *p* = 0.0031; 1.3|4.0 *p* = 0.0066; 1.1|2.9 *p* = 0.0333; 1.3|3.5 *p* = 0.0057, respectively). Approximately 7% of respondents thought veterinarians prescribed APs solely to make money, with the ones that gave APs to fish and reptiles having double odds of believing so (1.0|5.0 *p* = 0.0464 and 1.2|4.8 *p* = 0.0181, respectively).

## 4. Discussion

We start by indicating the limitations of this manuscript so the readers can have an appropriate assessment of the data stated in this study: in the construction of the questionnaire, we aimed for yes or no answers in order be able to more readily analyse them statistically; this type of answer was also more restrictive for respondents in indicating the limitations of the questions itself or their opinions on the subject. The fact that we used an open self-reporting survey made it impossible to calculate the response rate and the truthfulness or carefulness of the answers; also, in cases where the respondent is not certain of their answer, this may cause an overestimation of the overall results. Because we did not know the prevalence or NTCA ownership, we tried to predict our sample from the overall adult population. In the absence of a database of NTCA owner contacts and the usage of social media or emails, we induced a possible bias; thus, we were not able to achieve a random selection of participants.

### 4.1. Demographics

Having said that, the higher prevalence of answers from PT than SP is also observed in studies in veterinarians [61,62] and dog or cat owners [27,58]. The fact that the majority of respondents were from urban areas is in agreement with the literature [14]. The prevalence of respondents diminishes as the age increases, also agreeing with the other studies [6,7], but we should consider the bias from the methodology (using electronic forms and social media may reduce the reach to older respondents). In this study, we had a higher prevalence of female owners than a survey on endoparasitic infection risk in dogs or cats made in Spain [59]. It was not possible to determine whether the high prevalence of the upper class is a socioeconomic bias or if it reflects the actual predominance amongst these owners.

### 4.2. NTCA Numbers and Trade

The fact that a small percentage of respondents were breeders or sellers of NTCAs corroborates the idea that the keeping of these animals is now open to the wider population and not only the collector or hobbyist [4]; however, there was a greater prevalence of high-social-class respondents with NTCAs which may be related to the relatively high cost of maintaining these animals [13]. Around 45% of breeders were not registered as such, adding to the knowledge that owners are encouraged to buy from legitimate breeders and sellers [6] that are perceived by owners as trustable sources of information [12], and that animals kept illegally are less likely to seek veterinary advice [13], which may raise concerns about public health and the welfare of these animals [18].

Official data related to the trade of NTCAs in Europe are difficult to obtain [12,18], and when asked by the authors, government agencies were not helpful in shedding some light on the reality of NTCAs in PT and SP. So, adding our results of the prevalence of selected animal groups amongst owners to FEDIAF facts and figures of NTCA numbers in PT and SP may help to better understand the reality [8]. Although there is currently work being carried out to legislate the keeping of these animals [11,12], our results show that there is a lack of enforcement and knowledge of current legislation, agreeing with a study made in PT and the Eurogroup for Animals [5,12].

A big portion of respondents claimed to have conducted research on the species prior to the acquisition, but this was a self-evaluating report, and we did not evaluate the information itself. In other studies, it was hypothesised that owners may have the wrong idea about housing and nutrition perpetuated by general misconceptions [24,25], so further studies evaluating the sources or quality of information are needed so we can redirect owners to reliable information.

### 4.3. Veterinary Care

Regarding access to veterinary care, around 40% of respondents stated they have attended a ZM consult in the past, which is lower than described in Ireland, for example [2]. Although socioeconomic status and local availability were described as demographic factors influencing access to veterinary care [19], in our results, there was no significant difference in those factors.

### 4.4. Zoonotic Risks

When asked if respondents knew what a zoonosis was, 60% replied positively. These results agree with a complementary study made in PT for owners of dogs or cats [27], but we had different results in the influencing factors. Although we did not have any follow-up questions, it would be interesting to determine if they really knew what the meaning of the term was and if they would talk about it with their veterinarian or physician, as it was described in other studies [21,27,36].

### 4.5. Antibiotics

In one Australian study, the majority of owners did not think that ABs should be used, whether it was a viral or bacterial infection [44]; in our results, the equivalent proportion of respondents stated that ABs were used against bacteria, but around 24% also thought that they were used against fungus, parasites, or virus, although that percentage was lower in this study than in Portuguese undergraduates [63]. Additionally, in this study, the three most prevalent animal groups where ABs were used were similar when compared to the groups submitted for microbiological analysis in the Iberian peninsula [38].

The majority of respondents knew what AMR meant and its importance to human deaths, in line with other studies [46,63]. The percentage of respondents who think that giving their animal ABs can have a negative effect on them is in line with the results described by Scarborough et al. [44], but our results have a lower percentage of owners who felt that their animal needed ABs whenever they became sick. Our results also reinforce the idea that some owners may self-diagnose and wave off consulting their veterinarian [2,13] because a small percentage think that they know when their animal needs ABs or they feel that they could give ABs when they have the same signs, although almost all respondents claimed that prescribing ABs is a decision that should be made by the veterinarian. Almost all respondents claimed that they completed the course of ABs prescribed by their veterinarian, which is a bigger percentage than observed in other studies [46,63]. However, the percentage of owners who gave ABs without a veterinary prescription was similar to the lowest percentages in groups described by Marta-Costa et al. but more than three times lower than Candellone et al. [46,63]. The major source of ABs was the same as that described by Candellone et al. but in a lower proportion [46].

### 4.6. Parasiticides

There are some studies on deworming practices in dogs and cats in the Iberian Peninsula [27,58,59], but the authors did not find any study on NTCAs specifically worldwide. Nevertheless, our results show that a big part of owners acknowledge and accept faecal examinations when approached by their veterinarian; also, a large part of owners claim that they have talked with their veterinarian about an individual plan for AP usage, in line with current guidelines on the use of APs [26,50,52,57,59]. A significant percentage of respondents claimed they know when their animals need APs, which is in line with the premise by Carrasco [13]. Interestingly, some claimed they felt better when an AP was prescribed, and even more respondents felt safer if it was a broad-spectrum action one. This is in accordance with being a key factor for choosing APs [60]; however, it is contrary to current recommendations [52]. Favourably, our results showed that the majority of owners said they completed the course of APs prescribed, answering to the goals of better owner compliance set by Iberian studies [26,27,58,59].

## 5. Conclusions

The data presented here brought to light some topics that were, until now, anecdotal or perceptions at best. The answers given by owners show that there is still a need to improve education and registration of the NTCA trade because there is still a substantial part of unregistered or undocumented NTCAs. Even in the aftermath of a global pandemic, the ability of NTCA owners to recognise the term zoonosis was just over half of the respondents, which tells us that veterinarians and physicians should keep educating the general public on the subject. Also, people who did recognise the term zoonosis were more likely to acknowledge AMR and the inadequate use of APs. Although, overall, there are some good results on public perception of AMR or inadequate use of APs, there is still room for improvement. The information gathered here should help ZM veterinarians be more efficient in educating NTCA owners on multidrug resistance and zoonotic risks.

## Figures and Tables

**Table 1:** Cross-action between selected variables and recognising the term “zoonosis”.

Replied Yes to:	Recognises “Zoonosis”
Kept amphibians	77% 2.4 OR (1.6|4.3) ***
Kept birds	66% 2.1 OR (1.5|3.0) ***
Kept invertebrates	84% 4.2 OR (2.2|8.2) ***
Kept mammals	62% 1.8 OR (1.1|3.1) *
Kept fish	67% 2.1 OR (1.5|2.9) ***
Kept reptiles	65% 1.5 OR (1.0|2.1) *
Was a health professional	92% 13.2 OR (7.3|23.4) ***
Had a bachelor’s degree	68% 2.4 OR (1.7|3.5) ***
Investigated before acquiring	65% 2.6 OR (1.7|4.1) ***

OR—odds ratio; *—significant (*p* < 0.05); ***—highly significant (*p* < 0.001).

**Table 2:** Cross-action between selected variables and using antibiotics in determinate groups of animals.

Replied Yes to:	Used ABs in Amphibians	Used ABs in Birds	Used ABs in Fish	Used ABs in Invertebrates	Used ABs in Mammals	Used ABs in Reptiles
Was a breeder	13% 4.0 OR (1.7|9.4) ***	62% 4.4 OR (2.6|7.5) ***	NS	4% 7.1 OR (1.4|35.9) *	NS	36% 3.0 OR (2.2|6.8) ***
Was a seller	18% 5.2 OR (1.9|14.0) ***	65% 4.4 OR (2.1|9.0) ***	24% 4.1 OR (1.7|9.8) *	6% 7.9 OR (1.4|44.5) *	NS	41% 4.3 OR (2.1|8.9) ***
Was a health professional	9% 3.8 OR (1.7|8.5) ***	43% 2.0 OR (1.4|3.0) ***	NS	NS	NS	25% 2.5 OR (1.6|4.1) ***
Recognised the term “zoonosis”	8% 17.9 OR (2.4|133.2) ***	42% 3.4 OR (1.4|3.0) ***	12% 10.0 OR (3.0|32.6) ***	NS	80% 1.6 OR (1.1|2.4) *	21% 3.7 OR (1.9|6.0) ***
Recognised the term “AMR”	NS	NS	NS	NS	78% 3.5 OR (1.3|8.9) *	NS

AMR—antimicrobial resistance; OR—odds ratio; NS—nonsignificant; *—significant (*p* < 0.05); ***—highly significant (*p* < 0.001).

**Table 3:** Cross-action between selected variables, recognition of the term “AST”, and acceptance when proposed to caretakers.

Replied Yes to:	Recognises the Term “AST”	Accepted AST When Proposed
Kept amphibians	60% 2.0 OR (1.3|3.2) **	NS
Kept birds	NS	NS
Kept invertebrates	68% 2.8 OR (1.7|4.8) ***	NS
Kept mammals	48% 2.0 OR (1.1|3.5) *	NS
Kept fish	53% 2.0 OR (1.4|2.8) ***	NS
Kept reptiles	NS	NS
Was a breeder	58% 1.8 OR (1.1|2.9) *	NS
Was a seller	68% 2.6 OR (1.3|5.5) *	NS
Was a health professional	67% 3.7 OR (2.5|5.5) ***	72% 1.6 OR (1.1|2.4) *
Recognised the term “zoonosis”	60% 4.7 OR (3.2|6.9) ***	NS
Recognised the term “AST”	100% ***	84% 5.5 OR (3.6|8.2) ***

OR—odds ratio; NS—nonsignificant; *—significant (*p* < 0.05); **—very significant (*p* < 0.01); ***—highly significant (*p* < 0.001).

**Table 4:** Cross-action between selected variables and using parasiticides in determinate groups of animals.

Replied Yes to:	Used APs in Amphibians	Used APs in Birds	Used APs in Fish	Used APs in Invertebrates	Used APs in Mammals	Used APs in Reptiles
Was a breeder	20% 10.7 OR (4.6|24.7) ***	64% 4.7 OR (2.8|8.0) ***	35% 2.8 OR (1.5|5.3) **	9% 11.1 OR (3.1|40.1) ***	NS	38% 3.4 OR (2.0|5.9) ***
Was a seller	24% 8.9 OR (3.5|22.4) ***	71% 5.8 OR (2.7|12.4) ***	35% 4.6 OR (2.1|9.8) ***	12% 11.1 OR (3.0|41.6) **	NS	44% 4.1 OR (2.0|8.4) ***
Was a health professional	NS	48% 2.1 OR (1.5|3.2) ***	NS	NS	NS	28% 2.5 OR (1.6|3.9) ***
Recognised the term “zoonosis”	100% ***	42% 3.8 OR (2.5|5.8) ***	17% 4.3 OR (2.1|8.6) ***	NS	84% 1.6 OR (1.0|2.4) *	24% 3.5 OR (2.0|6.1) ***
Recognised the term “inappropriate use of AP”	6% *	35% 1.9 OR (1.2|3.1) **	NS	NS	85% 2.9 OR (1.8|4.7) ***	21% 4.9 OR (2.1|11.5) ***
Went to a ZM appointment	NS	37% 1.5 OR (1.0|2.1) *	NS	NS	NS	22% 1.6 OR (1.1|2.6) *

APs—parasiticides; ZM—Zoological Medicine; OR—odds ratio; NS—nonsignificant; *—significant (*p* < 0.05); **—very significant (*p* < 0.01); ***—highly significant (*p* < 0.001).

**Table 5:** Cross-action between selected variables, recognition of the term “coprological analysis”, and acceptance when proposed to caretakers.

Replied Yes to:	Recognises the Term “CA”	Accepted CA When Proposed
Kept amphibians	85% 1.9 OR (1.1|3.5) *	NS
Kept birds	79% 1.7 OR (1.1|2.5) *	NS
Kept invertebrates	NS	NS
Kept mammals	79% 2.5 OR (1.5|4.4) **	82% 2.3 OR (1.3|4.1) **
Kept fish	NS	NS
Kept reptiles	NS	NS
Was a breeder	91% 3.6 OR (1.5|8.6) **	NS
Was a seller	97% 10.9 OR (1.5|80.6) **	NS
Was a health professional	88% 3.0 OR (1.8|5.0) ***	85% 1.7 OR (1.0|2.7) *
Recognised the term “zoonosis”	88% 4.8 OR (3.1|7.4) ***	85% 2.2 OR (1.4|3.3) ***
Recognised the term “CA”	100% ***	90% 11.1 OR (6.9|18.0) ***

CA—coprological analysis; OR—odds ratio; NS—nonsignificant; *—significant (*p* < 0.05); **—very significant (*p* < 0.01); ***—highly significant (*p* < 0.001).

## Data Availability

The datasets generated and/or analysed during the current study are available from the corresponding author upon reasonable request.
